# Hemolytic uremic syndrome with simultaneous Shiga toxin producing *Escherichia coli* and complement abnormalities

**DOI:** 10.1186/1471-2431-14-278

**Published:** 2014-11-05

**Authors:** Nicole McCoy, Donald J Weaver

**Affiliations:** Department of Pediatrics, Levine Children’s Hospital at Carolinas Medical Center, Charlotte, NC 28232 USA; Division of Nephrology and Hypertension, Levine Children’s Hospital, 1001 Blythe Boulevard, Ste 200, Charlotte, NC 28232 USA

**Keywords:** Hemolytic uremic syndrome, Atypical hemolytic uremic syndrome, Acute kidney injury, Shiga toxin-producing *E.coli*, Complement factor H, Therapeutic plasma exchange

## Abstract

**Background:**

Hemolytic uremic syndrome is a common cause of acute kidney injury in children. In children, hemolytic uremic syndrome is most commonly associated with gasterointestinal infections caused by Shiga toxin-producing *Escherichia coli* or other enteric organisms. Although less common, atypical hemolytic uremic syndrome is triggered by multiple factors and portends a significantly worse prognosis with a high rate of recurrence.

**Case presentation:**

Here we discuss the case of a 10 year old Caucasian male presenting with thrombocytopenia, anemia, and acute kidney injury.

**Conclusions:**

This case highlights the clinical challenges in diagnosing and managing patients with hemolytic uremic syndrome. Because of similarity in symptoms, differentiating Shiga toxin-producing *Escherichia coli* associated hemolytic uremic syndrome and atypical hemolytic uremic syndrome can be challenging. However, because of the increased morbidity and mortality of atypical hemolytic uremic syndrome, early detection and initiation of therapy are critical. Providers must have a heightened suspicion in order to initiate supportive care or disease directed therapy in the case of atypical hemolytic uremic syndrome.

## Background

Hemolytic uremic syndrome (HUS) is a systemic disease manifested by acute kidney injury, thrombocytopenia, and hemolytic anemia. In fact, HUS continues to be cited as the most common cause of acute kidney injury in young children [1]. Generally, HUS is associated with gasterointestinal infections caused by Shiga toxin-producing *Escherichia coli* (STEC) or other enteric organisms [1]. In the acute phase of the illness, two-thirds of patients require renal replacement therapy with a majority of those patients experiencing renal recovery [1]. The mortality rate in STEC HUS is between 3-5% (1). As only 10-15% of children infected with STEC progress to HUS, it is hypothesized that an underlying genetic predisposition or additional environmental stimuli may be involved [1]. Non-STEC causes of HUS or atypical HUS (aHUS) are typically associated with mutations in regulatory proteins of the complement system in children. More importantly, aHUS is associated with increased morbidity and mortality. Current treatment strategies for HUS generally focus on supportive care including stabilization of the renal and hematologic manifestations. Plasmapheresis, infusion of fresh frozen plasma, and fibrinolytic therapy have been proposed to have theoretical benefit, but based on a recent Cochrane review, there is no evidence to support these interventions at this time [2, 3]. However, in aHUS, disease-directed therapy is available but must be initiated quickly to prevent significant sequelae.

## Case presentation

A 10-year old male presented to an urgent care center following 24 hours of non-bloody diarrhea and abdominal pain. He was diagnosed with viral gastroenteritis and oral rehydration was recommended. On day 5 of illness, his symptoms improved, but by day 8, he developed increasing listlessness and lethargy. By day 9 of illness, the patient developed non-bloody, non-bilious emesis and was re-evaluated at the urgent care center. He was again diagnosed with viral gastroenteritis although laboratory studies were obtained prior to discharge. The parents were notified on day 12 of illness that these studies were significantly abnormal. The patient was brought to the local emergency room and admitted to the Pediatric ICU for further management.

**Figure 1:**
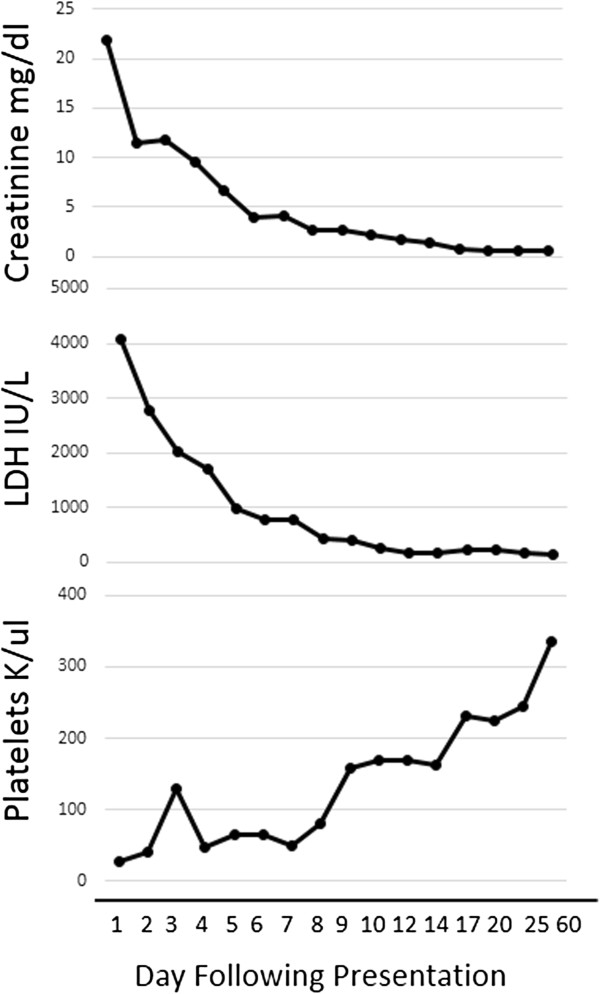
**Improvement in laboratory parameters upon initiation of therapeutic plasma exchange.** Daily therapeutic plasma exchange was initiated on hospital day 4. The patient was then transitioned to alternate day therapeutic plasma exchange. Plasma exchange was discontinued on day 50.

Upon arrival, the patient was lethargic and confused. His vital signs demonstrated tachycardia and hypertension. His pupils were round, equal and reactive to light bilaterally. He had bloody crusting at nares bilaterally. His respiratory, cardiac, abdominal, and musculoskeletal exams were normal. His skin exam demonstrated jaundice and bruising on his legs. Despite difficulty focusing and communicating, he did have purposeful movements with painful stimuli and bilateral ankle clonus. Laboratory studies confirmed a hemoglobin of 4.5 g/dl and a platelet count of 86 K/ul. His basic metabolic panel revealed a sodium of 124 mg/dl, a potassium of 6.0 mg/dl, a bicarbonate of 13 mg/dl, a blood urea nitrogen of 329 mg/dl, and a creatinine of 21.8 mg/dl (Figure 1). Additional labs included a LDH of 4067 IU/L and a PTT of 27 seconds. A chest radiograph was obtained and was remarkable for pulmonary edema.

**Figure 2:**
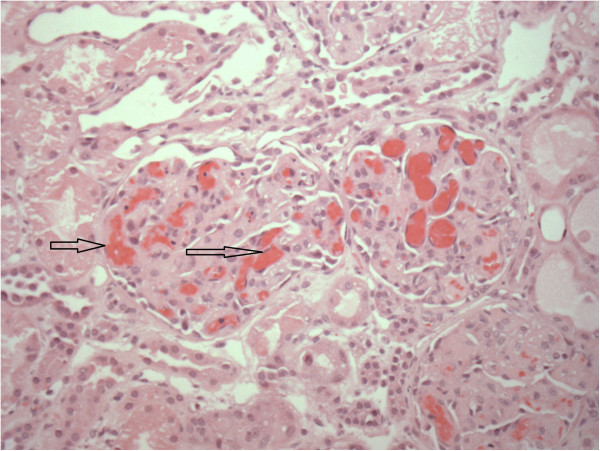
**Hematoxylin and eosin stain of glomeruli showing red cell sludging and thrombi formation (arrow) typical of hemolytic uremia syndrome.**

**Figure 3:**
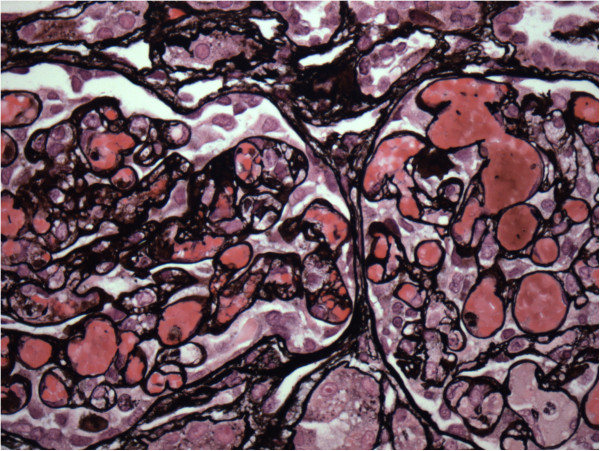
**Jones stain showing fibrin thrombi and vascular congestion of glomeruli suggestive on hemolytic uremia syndrome.**

Following platelet and packed red blood cell tranfusions because of the potential for bleeding, a temporary external jugular hemodialysis catheter was placed and hemodialysis was initiated. A renal ultrasound was normal. A percutaneous renal biopsy was performed on hospital day 3 to confirm the diagnosis and to examine the degree of chronicity because of the severity of acute kidney injury. Results from the biopsy demonstrated the presence of a thrombotic microangiopathy on hospital day 4 (Figures 2 and 3). Because of the severity of his illness and concern for atypical causes of HUS, therapeutic plasma exchange (TPE) was initiated on hospital day four. Laboratory studies at that time revealed a normal complement C3 level, a normal complement C4 level, and a negative anti-neutrophil antibody titer. ADAMTS 13 activity was also normal at 40%. Once stool was available on hospital day 6, a sample was obtained for bacterial culture and to assess for Shiga-like toxin using enzyme-linked immunoabsorbent assay simultaneously. After 48 hours, stool culture demonstrated no bacterial growth including no evidence of *Escherchia coli* O157:H7 on Sorbitol-MacConkey. Genetic studies for mutations in complement system regulatory proteins were also obtained. He was extubated on day 6. The patient was maintained on daily TPE for five sessions and transitioned to alternate day TPE on hospital day 9 with stabilization of his renal function and hematologic studies (Figure 1). Hemodialysis was discontinued on hospital day 10 (Figure 1). The patient was discharged on hospital day 12 and continued to receive alternate day TPE as an outpatient (Figure 1). Laboratory studies at discharge included a BUN of 26 mg/dl, serum creatinine of 1.71 mg/dl, hemoglobin on 7.1 g/dl, platelets of 170 k/L, and a LDH of 262 IU/L. Following discharge, a stool sample was positive for E. coli Shiga like toxin based on enzyme immunoassay. Based on this result, the frequency of TPE was decreased and ultimately discontinued on day 50 following his diagnosis. His laboratory studies remained normal upon discontinuation of TPE. However, results from genetic testing obtained while hospitalized returned 3 months following his illness revealed mutations in regulatory components of the alternative complement pathway including a heterozygous missense mutation in complement Factor H (Gln950His) as well as a homozygous deletion in complement factor H-related genes, *CFHR1* and *CFHR3*. Autoantibody to Factor H was negative.

Despite discontinuation of therapy and his initial renal dysfunction, the patient continued to maintain normal electrolytes, normal hematologic studies, normal renal function, normal blood pressure, and a normal urinalysis. Therefore, TPE was not restarted. A year following his initial diagnosis he has not had any recurrence of his disease, and laboratory studies are remarkable for a serum creatinine of 0.47 mg/dl, platelets of 381 K/uL, and LDH of 182 IU/L.

## Conclusions

In the case discussed above, the patient demonstrated a prodromal illness of loose stools and emesis with subsequent development of thrombocytopenia, anemia, and acute kidney injury consistent with STEC HUS. However, because of the severity of his renal dysfunction and lack of definitive evidence for STEC on stool culture, TPE was initiated. Anecdotally, TPE has purported benefits in patients with STEC HUS although a recent meta-analysis argued against these conclusions [2]. Another consideration for initiation of TPE was the possibility of aHUS which is associated with alterations in regulatory factors of the complement system [4]. These mutations lead to unregulated activation of the alternative complement pathway, activation of the membrane attack complex, and endothelial damage [4]. Although less frequent, aHUS has a poor prognosis with 50% of cases progressing to end stage renal disease (ESRD) and a mortality rate of 25% during the initial episode [4]. In addition, aHUS has a high risk of recurrence [4]. As patients with aHUS can often have non-specific gasterointestinal complaints, the distinction between the presentations of patients with STEC HUS compared to patients with aHUS is often difficult [4]. Unfortunately, definitive genetic testing is only performed at a few laboratories in North America and often takes 3-4 months. Most importantly, treatment strategies to prevent this morbidity and mortality are available including plasma exchange, plasma infusion, as well as a humanized monoclonal antibody (Eculizumab) that blocks activation of the terminal complement pathway [3]. Therefore, providers must maintain a high index of suspicion and initiate therapy often in the absence of confirmatory testing [4]. As illustrated above, the patient demonstrated a rapid improvement in both hematologic and renal parameters upon initiation of TPE. But 9 days into treatment, confirmatory testing for Shiga-toxin was positive on enzyme immunoassay (EIA) suggesting that STEC triggered the development of HUS [5]. Recent guidelines suggest that simultaneous culture for O157 STEC and EIA testing for Shiga toxin is more effective at detecting STEC than each method alone for several reasons including bacteria are difficult to detect after 1 week of illness and non O157 STEC may be causing disease [5]. As a result, the patient’s plasma exchange therapy was gradually weaned, and his hematologic parameters as well as his renal function remained normal.

Unexpectedly, genetic studies obtained at the time of presentation and resulted three months later indicated that the patient possessed mutations in complement regulatory factors that are associated with development of aHUS specifically complement factor H (CFH) and as well as a homozygous deletion in complement factor H-related genes [6, 7]. CFH is a critical regulator of the alternative complement cascade preventing generation of terminal complement components and the membrane attack complex [6]. Mutations in CFH are the most commonly identified genetic abnormality in patients with aHUS. In fact, the mutation noted in the current patient has been associated with the development of aHUS in the literature and is thought to alter the C-terminal domain of CFH [8]. Mutations in CFHR1/CFHR3 are found in 32% of patients with aHUS and associated with development of CFH antibodies which is a risk factor for the development of aHUS [7]. The current patient did not demonstrate CFH autoantibodies. In the absence of CFH autoantibodies, the significance of deletions of CFHR1/CFHR3 with a simultaneous mutation in CFH has not been described. Interestingly, incomplete penetrance is reported for all of the genes associated with aHUS [8]. Even in patients with mutations in two genes associated with aHUS, penetrance is still incomplete. These data suggest that development of the clinical findings of aHUS is dependant on other genetic risk factors and environmental events [9]. Specifically, 70% of cases of aHUS with mutations in CFH are preceded by an infection [6]. Pregnancy and medications are associated with precipitation of the clinical manifestations of aHUS in 8% of cases [6]. Therefore, in concert with mutations in complement regulatory factors and other genetic risks, an environmental trigger is required to initiate the unregulated activation of the complement system and development of HUS [10]. In the patient described above, the environmental trigger was hypothesized to be Shiga toxin-mediated damage of the endothelial cell. The presence of these genetic risk factors may have also influenced the severity of his renal presentation.

The clinical implications of these results suggested that as opposed to a majority of patients with STEC-associated HUS, the patient described above is at risk for recurrence of his disease [1, 4]. A recent article describing a cohort of patients with aHUS found that the risk of recurrence is approximately 40% which decreases to 25% after the first year [11]. Therefore, close monitoring of laboratory studies remains critical in these patients particularly during times of acute illness. Furthermore, with the development of novel therapies that seem to block complement-mediated damage associated with aHUS, the clinical dilemma becomes when to initiate directed therapy for HUS as early intervention significantly improves clinical outcomes [11, 12]. At present, the patient described above does not demonstrate any signs of recurrent disease. Despite his initial serum creatinine of 21 mg/dl, he maintains normal renal function, normal hematologic parameters, normal blood pressures, and normal urinalysis. Therefore, there is no indication for disease-specific therapy at this time. However, the patient’s family has been counseled extensively on the importance of obtaining laboratory studies at signs of fever, illness, or other factors associated with a recurrence of HUS so that therapy could be initiated as early as possible.

In summary, this case highlights the clinical challenges in managing patients with HUS. Despite recent advances in the pathophysiology of STEC HUS, several issues remain including identifying the genetic and environmental factors that confer susceptibility for development of HUS in select patients following infection with STEC. In addition, because the symptoms of STEC associated HUS overlap with symptoms noted in patients with aHUS, providers must have a heightened suspicion in order to initiate supportive care or disease directed therapy in the case of aHUS. Finally, additional research is required to determine if a subset of patients with STEC HUS such as the patient described above may benefit from solely supportive care or disease specific therapy.

## Consent

Written informed consent was obtained from the patient’s parents for publication of this case report and any accompanying images. A copy of the consent form is available for review by the Editor of this journal.

## Abbreviations

HUS: Hemolytic uremic syndrome
STEC: Shiga toxin producing Escherichia coli
TPE: Therapeutic plasma exchange
aHUS: atypical hemolytic uremic syndrome
LDH: Lactate dehydrogenase
CFH: Complement factor H.
